# Development and Experimental Research of Different Mechanical Designs of an Optical Linear Encoder’s Reading Head

**DOI:** 10.3390/s22082977

**Published:** 2022-04-13

**Authors:** Donatas Gurauskis, Krzysztof Przystupa, Artūras Kilikevičius, Mikołaj Skowron, Jonas Matijošius, Jacek Caban, Kristina Kilikevičienė

**Affiliations:** 1 Institute of Mechanical Science, Vilnius Gediminas Technical University, J. Basanavičiaus Str. 28, LT-03224 Vilnius, Lithuania; donatas.gurauskis@vilniustech.lt (D.G.); arturas.kilikevicius@vilniustech.lt (A.K.); jonas.matijosius@vilniustech.lt (J.M.); kristina.kilikeviciene@vilniustech.lt (K.K.); 2 Department of Automation, Lublin University of Technology, Nadbystrzycka 36, 20-618 Lublin, Poland; j.caban@pollub.pl; 3 Department of Electrical and Power Engineering, AGH University of Science and Technology, A. Mickiewicza 30, 30-059 Krakow, Poland

**Keywords:** optical encoder, reading head, measuring scale

## Abstract

Optical linear encoders are widely used in manufacturing. They are accurate and have a relatively high resolution and good repeatability. However, there are a lot of side effects, which have an inevitable impact on the performance of an encoder. In general, the majority of these effects could be minimized by the appropriate design of an encoder’s reading head. This paper discusses the working principle of and commonly occurring errors in optical linear encoders. Three different mechanical designs are developed and implemented in the experimental reading head of the linear encoder in order to evaluate how mechanical construction influences the displacement measurement accuracy and total performance of the encoder.

## 1. Introduction

An encoder is a device that transforms linear or angular mechanical motion into electrical signals. According the nature of this mechanical motion, encoders are classified as linear [1] and rotary [2]. There are various displacement measurement technologies based on different physical phenomena, such as optical, magnetic [3,4,5], inductive [6,7], and capacitive [8,9,10]. Optical linear encoders are a dominant type of sensors in the high-resolution market. They are insensitive to a magnetic field compared with magnetic encoders and have a high resolution, accuracy, and repeatability. In general, the working principle of optical encoders is based on light modulation during a relative movement of two gratings. Displacement could be measured by using different optical effects and principles, such as the Talbot effect [11,12], the Lau effect [13,14], generalized grating imaging [15,16,17,18], interferometry [19,20,21], and the Moiré effect [22,23,24].

Linear encoders with an optical scanning method are the most widespread choice for CNC machines and common machine tools. Optical gratings of 10–20 microns are usually used, and a resolution of around 100 nm could be reached. However, there are a lot of side effects, which have an inevitable impact on measurement accuracy, repeatability, possible resolution, and total performance. Various deformations and translations of optical and other components [25], mechanical vibrations [26,27,28], changes in temperature [29,30,31], variation in electrical signals’ amplification, filtration, or interpolation could occur while an encoder works under real environmental conditions. However, the majority of these unwanted effects could be avoided or drastically reduced by the appropriate design of the encoder’s reading head. The scientific literature provides a lot of information, and a number of studies have been performed in the field of optical scanning or the monitoring and handling of electrical signals. However, little is known about the direct influence of mechanical construction itself. It is important to bear in mind that components of even the most advanced optical scanning system are integrated into the mechanical design of the reading head, which itself can generate additional measurement error. Development and design of the encoder and its parts are usually based on the practical knowledge of the manufacturer. There are a lot of possible engineering solutions and various mechanical designs suitable for such a displacement measurement device.

Manufacturers provide for the optical linear encoder’s performance under normative conditions. Accuracy, repeatability, and resolution of optical linear encoders are indicated at a normative temperature 20 °C and under laboratory environmental conditions. In real applications of optical linear encoders occur dynamic (i.e., time-varying, temperature-varying) factors that cause dynamic errors. Dynamic error components are not evaluated in optical linear encoder documentation. In most cases, when operating high-precision devices (such as CNC and CMM), dynamic factors are compensated by evaluating an entire mechatronic or robotic system with integrated optical linear encoders. For this type of compensation, optical linear encoders are accepted as ideal measurement systems that operate without dynamic errors.

**Figure 1:**
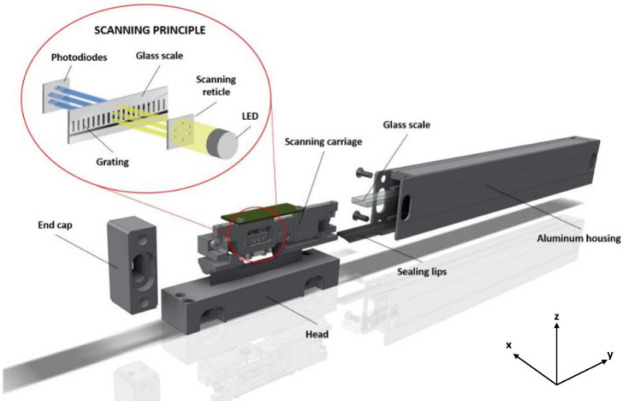
A scheme of the basic principle of the typical optical linear encoder.

The typical optical linear encoder consists of an aluminum extrusion with a glass scale and a reading head. The reading head is fitted with the scanning carriage, which holds optical elements such as an LED, a scanning reticle, photodetectors and other electronic components, and the housing. A flexible spring-based suspension is used to connect the housing with the carriage. Such a suspension must not only be flexible and absorb all the deviations of the application guideway but also limit additional motion of the scanning carriage along the glass scale. To achieve this, some rigid parts, such as hardened steel plates and pins, must be kept in contact during the bi-directional motion of the reading head. High acceleration movements, mechanical vibrations, or friction between protecting lips and the reading head could cause a temporary loss of contact, so stable contact between these mechanical parts (referred to as “a contact pair”) is especially important. The typical linear encoder is shown in Figure 1.

The objective of this paper is an experimental investigation of the influence of different types of mechanical designs on the operation of a linear encoder. To perform tests and evaluate reliability and resistance [32,33,34] to various working conditions, an experimental reading head with three different possible mechanical constructions (contact pairs) has been developed. One of them is close to a solution used in widespread linear optical encoders, and the other two are newly designed contact pairs with their own advantages, such as selected mechanical components and a simple assembly process. All three contact pairs are integrated into a single experimental reading head, but only one contact pair is used at a time. Such an approach allows us to maintain the same optical and electronic components and reduce the uncertainty of the tests.

## 2. Optical Linear Encoders

### 2.1. Working Principle

**Figure 2:**
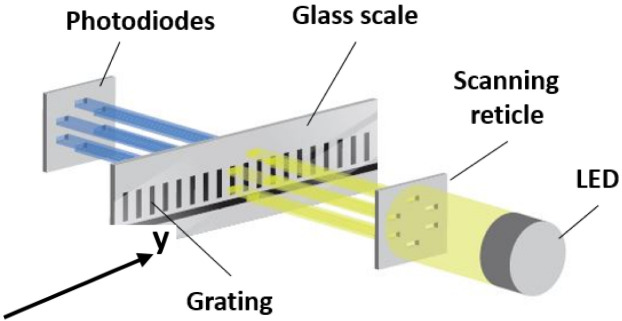
Optical principle of Moiré-based optical linear encoders.

The working principle of an optical encoder is commonly based on the light modulation principle, as shown in Figure 2. In such an optical configuration, a well-collimated infrared light beam spreads through a system, formed by two gratings of the same period *p*. The first grating is placed on the reference scale, usually made of glass, and the other one on the so-called scanning reticle. The grating of the reticle is divided into four fields for electrical signal generation and two additional fields for reference mark. A relative positioning of the fields (0°, 90°, 180°, and 270° degrees of period) allows us to generate near-sinusoidal signals with a different phase shift. The total measuring range of an encoder is defined by the length of this scale. When modulated light passes both of the gratings, its intensity *I* is sensed by the array of photodiodes. Any displacement between two precisely aligned gratings produces a variation in the light intensity signal that has a fundamental frequency of 1/*p* and additional harmonics.

Correctly aligned photodiodes under the scanning reticle fields are used to generate electrical signals [35].

(1) $$I_{0^{{}^{\circ}}}=a+b\;cos\left(2\pi \frac{y}{p}\right)$$

(2) $$I_{90^{{}^{\circ}}}=a+b\;cos\left(2\pi \frac{y}{p}+\frac{\pi }{2}\right)=a-b\;sin\left(2\pi \frac{y}{p}\right)$$

(3) $$I_{180^{{}^{\circ}}}=a+b\;cos\left(2\pi \frac{y}{p}+\pi \right)=a-b\;cos\left(2\pi \frac{y}{p}\right)$$

(4) $$I_{270^{{}^{\circ}}}=a+b\;cos\left(2\pi \frac{y}{p}+\frac{3\pi }{2}\right)=a+b\;sin\left(2\pi \frac{y}{p}\right)$$

where *a* is the irradiance bias, *b* is the modulation depth of the signal, and *y* indicates the position of the reading head.

The formed sinusoidal signals are electronically processed and interpolated to increase resolution and are used for a precise displacement measurement. The interpolated position within a period is then calculated using [36]:

(5) $$y=\frac{p}{2\pi }arctan\left(\frac{I_{270^{{}^{\circ}}}-I_{90^{{}^{\circ}}}}{I_{0^{{}^{\circ}}}-I_{180^{{}^{\circ}}}}\right)$$

This measurement process is directly related to the quality of electrical signals. They can be distorted by a number of side effects, such as the relative positioning of the glass scale and the scanning reticle, optical noise fluctuations, and mechanical vibrations.

### 2.2. Errors in Optical Linear Encoders

The quality of optical and other components as well as the interaction between them cause the formation of poor-quality electrical signals and are the source of high-frequency metrological errors that are repeated in each period of the measuring scale. Effects such as changing ambient temperature, mechanical deformation of the glass scale, or translation between the parts of the reading head could cause low-frequency measurement errors. The most common errors in optical linear encoders are discussed below [37].

#### 2.2.1. Optical Noise Fluctuations

**Figure 3:**
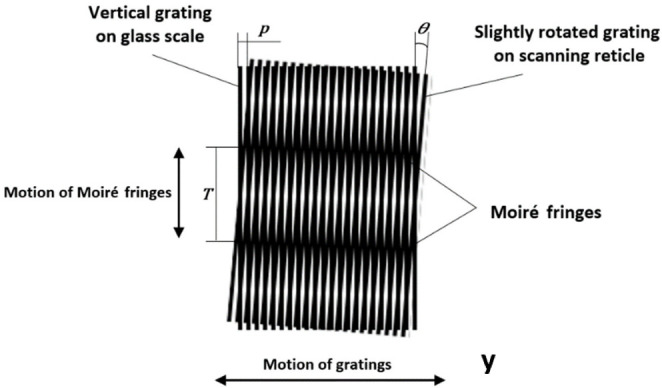
Superposition of the two slightly rotated gratings and the formation of Moiré fringes.

In practical applications, the superposition of straight-line grating structures leads to the formation of an interference pattern (Moiré fringes). The form, position, and pitch of such fringes directly depend on the reciprocal position and geometrical parameters of two gratings. The relevant combination of gratings has a magnification effect, and even a small displacement between the glass scale and the reading head of an encoder will lead to a much larger displacement of Moiré fringes [38]. The superposition of the two gratings and the formation of Moiré fringes are shown in Figure 3.

Optical noise fluctuation is directly related to imperfections in the measuring scale over its length. The grating formed of transparent and opaque bars is usually produced by using photolithography. For reflective-type encoders, gratings are formed on stainless-steel tape by using technologies such as electron beam or focus ion beam writing [38,39] and nanoimprint lithography [40].

In Moiré techniques, the sensitivity of displacement measurement increases with grating frequency. However, the fabrication of a high-resolution grating is a difficult task because of the technological process and material problems. Even a small deviation in a grating period affects the quality of measurement.

The opening ratio and the form of the gratings are also important and have a strong effect on electrical signals. The intensity difference between the minimum and the maximum values of the Moiré fringe pattern is the highest when the opening ratio of the measuring scale and the scanning reticle gratings is equal to 0.5. According to intensity distributions, a square-shaped grating generates an approximately triangular wave form and a sine grating a sine wave form. Considering the following processing of the Moiré profile, both grating forms could be adapted to use in linear encoders.

Unwanted optical fluctuations could also be generated by variation in light source intensity and sensitivity of the photodiodes. Non-perfectly collimated light also distorts Moiré patterns and leads to poor contrast. The quality of the used elements and precise alignment could help to avoid these harmful side effects.

#### 2.2.2. Relative Position between Optical Components

One of the most important things is to ensure a high contrast of Moiré fringes in order to achieve highly accurate measurement. In the real mechanical construction of an encoder, a finite size gap between gratings is introduced. Because of non-perfect collimated light and diffraction effects, which become more significant in gratings with a period of 100–200 μm, a variation in the gap has a major influence on fringe contrast [41,42].

Hane et al. [43] suggested the use of an optical configuration in which the second grating is mounted with an adjusted tilt. Ieki et al. [44,45] introduced a pitch-modulated phase grating. All these works are related to a reduction in the sensitivity to an air gap. It is sometimes hard to achieve such improvements in real applications because more space and special technological solutions are required.

Commonly, to not lose the contrast among Moiré fringes and to obtain good-quality signals, the air gap between the glass scale and the scanning reticle should be around 50 μm [16].

The formation and parameters of Moiré fringes depend on the superposition of two gratings. As shown in Figure 3, the scanning reticle is slightly rotated to form a certain angle *θ* between the gratings. Spacing *T* of the formed fringes could be expressed as [35]:

(6) T=p2sin(θ2)

Any changes in fringe spacing lead to an inappropriate formation of electrical signals because the photodetectors are aligned according to the location of the fringes. Even a small angle tilt between the gratings results in large displacement measurement errors in the encoder.

Song et al. [46,47] presented the phase-shifted grating to reduce tilt errors. A scanning reticle with a specially formed grating could be used to compensate the non-orthogonal error caused by a tilt of the index scale.

#### 2.2.3. Mechanical Deformation and Translation

Mechanical deformations of the glass scale could occur when the encoder is assembled or mounting proceeds incorrectly. Due to its comparably lower stiffness, the glass scale deforms and begins to repeat the irregularities of the aluminum extrusion. Certain additional deformation of the aluminum profile occurs due to the lack of application surface flatness or local deformations caused by fixing and clamping elements.

Translation between reading head parts is especially harmful because it can generate an unwanted relative motion of optical components, as already discussed for tilt and gap variation errors. The loss of contact between contact pair parts leads to a direct displacement measurement error because of redundant scanning carriage motion along the glass scale.

#### 2.2.4. Thermal Errors

Thermal errors mainly arise in the glass scale because its length changes if temperature changes. Along with that, a period of reference grating is altered. Mechanical expansion or contraction is also noticeable in other encoder parts. These thermoelastic effects become more difficult due to different thermal expansion coefficients of the aluminum housing of the encoder, the glass scale, and the rigid support of the application in which the encoder is mounted. Such a combination of assembled parts leads to difficulty in forecasting thermal behavior and false displacement measurement [29,30].

#### 2.2.5. Mechanical Vibration

The technological process of the system into which the optical encoder is integrated generates mechanical vibrations. If external excitation coincides with natural frequencies of encoder parts, it could cause temporal or permanent errors. The mechanical construction of the encoder must ensure high resonant frequencies. Even the type of mounting of the encoder [26] or the scanning principle [27] has a different influence on its performance under vibrations.

## 3. Development of the Experimental Reading Head

Basically, the reading head consists of two parts: a housing and a scanning carriage (cursor). The housing of the reading head is usually attached to a moving unit, while the aluminum encoder’s profile, with the glass scale, is usually fixed on a rigid support of the application. The cursor holds a scanning reticle and is preferably guided along the surface of the glass scale. If the housing or the reading head of the encoder is inaccurately mounted, or motion is not perfectly straight, a flexible spring-based suspension between the housing and the scanning carriage is required. Motion is correctly transmitted to the cursor if these two parts have a rigid contact point. Hardened or rigid stainless-steel parts, such as pins and grinded plates, must be kept in contact while measurement is processed in both directions. These parts could be maintained together by using the force of extension springs, a permanent magnet, etc.

### 3.1. Spring-Based Suspension

Proper cursor attachment and fluent motion along the surface of the glass scale are usually achieved by using small ball bearings. Three ball bearings are aligned on a single glass surface and another two on a perpendicular one. The scanning carriage is pushed toward the scale by a spring-based system. Such a suspension should protect the reading head from any mechanical damage and ensure a permanent connection between the cursor and the glass scale.

**Figure 4:**
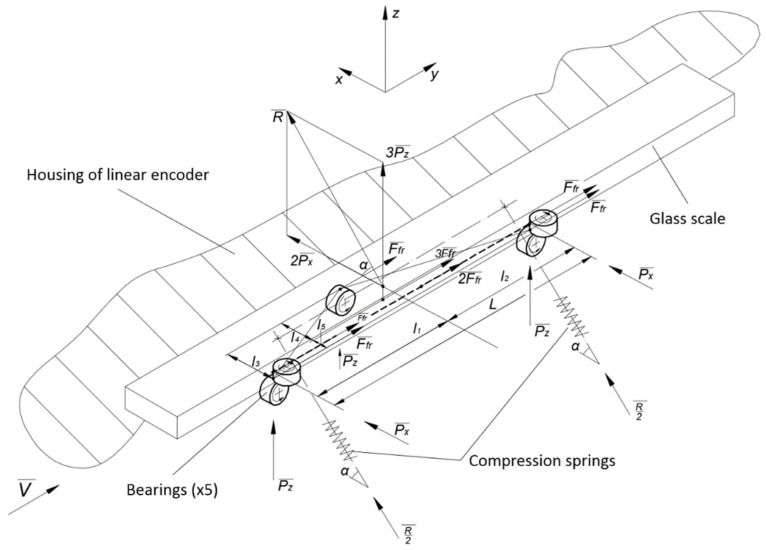
A scheme of the basic principle of a spring-based suspension of the experimental reading head.

This paper presents an experimental reading head with a flexible system based on two compression springs, positioned at a specific angle α. A scheme of its basic principle is shown in Figure 4.

In such a combination, there are five points of contact between the cursor and the glass scale. The forces acting in these points are marked as *P_x_* and *P_z_*, considering the specified coordinate axes. According to the alignment of the ball bearings, the resultant force *R* is acting toward the glass scale at an angle *α*. If all acting forces are equal, i.e., *P_x_* = *P_z_* = *P*, the resultant force *R* and the angle can be expressed by the following equations:

(7) $$R=\sqrt{{\left(2P_{x}\right)}^{2}+{\left(3P_{z}\right)}^{2}},\;\mathrm{if}.\;P_{x}=P_{x}=P\;\mathrm{then}\;R=P\sqrt{13}$$

(8) $$\alpha =arctan\left(\frac{3P_{z}}{2P_{x}}\right)\;\mathrm{if}\;P_{x}=P_{x}=P\;\mathrm{then}\;\alpha =56{}^{\circ}19’$$

**Figure 5:**
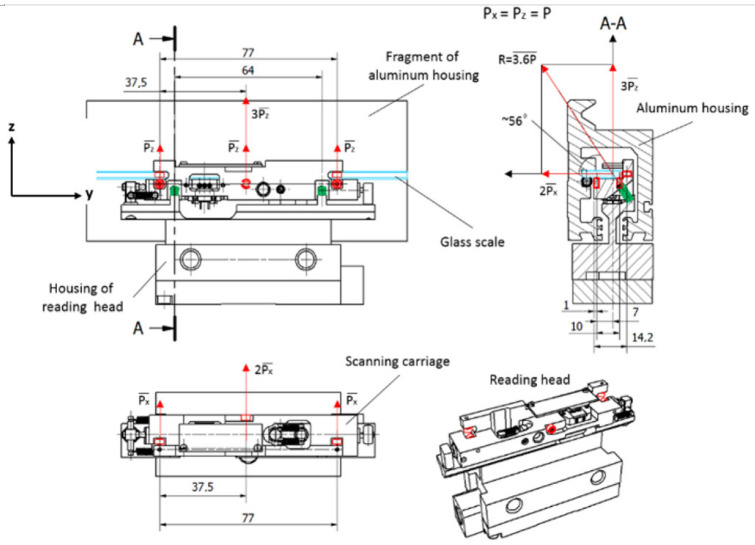
Mechanical construction of the reading head with the marked acting forces. (For better visualization, the glass scale is marked in the drawing in light blue, the compression springs in green, and the ball bearings and acting forces in red).

During the measurement, the reading head can move at high velocities and accelerations. It is important that the induced inertial forces do not exceed the resultant force. The mass of the scanning carriage with all its components is about 30 g. The declared permissible acceleration for this kind of encoders is ≤ 120 m/s^2^. Following Newton’s second law, the required force is calculated from the following equation:

(9) R=k(m×a)

where *m* is the mass, *a* is the permissible acceleration, and *k* is the factor of safety (*k* = 1.6). The calculated force *R* = 5.8 N is generated if two compression springs of ~5.6 N maximum load and 0.384 N/mm spring rate are embedded into the construction, positioned at ~56° angle *α*. This mechanical design is shown in Figure 5.

### 3.2. Rigid Contact between the Cursor and the Housing of the Reading Head

**Figure 6:**
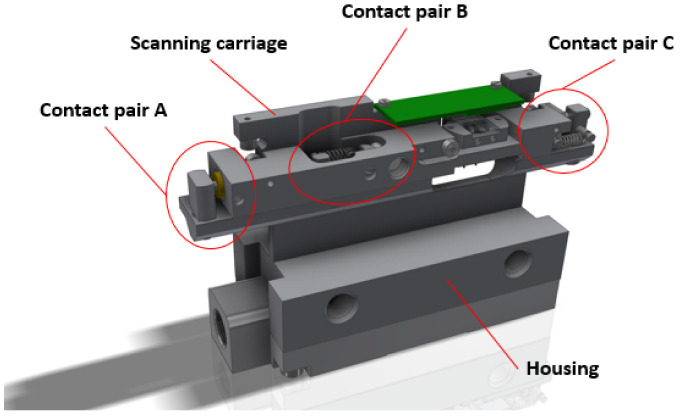
A 3D CAD model of the designed reading head with the marked contact pairs.

The housing of the reading head and the cursor are connected with springs, but they also must have rigid contact for exact motion. This connection must be maintained between two solid parts (so-called contact pair) in both measuring directions along the glass scale. Any deformations or translations in this contact point have a direct influence on the accuracy, repeatability, and stability of the encoder. The designed reading head has three different contact pairs, which can be easily disconnected and used only one at the time. The 3D CAD model with the marked arrangement of contact pairs A, B, and C is shown in Figure 6.

As shown in the calculation scheme (Figure 4), there are five friction forces *F_fr_* acting between the ball bearings and the surface of the glass scale. This side effect must be considered when the required force *S* is determined to keep the elements of the contact pair in touch. Using the calculated resultant force *R* value, the acting forces *P_x_* and *P_z_* can be calculated from Equation (8) (*P* = 1.6 N). The acting friction force is calculated as follows [48]:

(10) $$F_{fr}=k_{fr}\times P$$

where *k_fr_* is the coefficient of friction between the ball bearing and the polished glass surface (*k_fr_* = 0.003… 0.005). The necessary force value for the contact pair is calculated as follows:

(11) $$S\geq k\left(R+5F_{fr}\right)$$

The total value of the acting friction forces is relatively negligible. The required force *S* is approximately equal to 6 N. Similarly to the flexible suspension design, various solutions could be used to generate this force. Usually, it is achieved by extension springs or a force of the permanent magnetic field.

All designed contact pairs are arranged in different places of the experimental reading head and consist of various elements so that each of the designs can be compared by testing the total accuracy of the encoder.

#### 3.2.1. Contact Pair A

In contact pair A, the grinded steel plate and the hardened pin are kept in contact by the magnetic force of the permanent neodymium magnet. This construction is simple and easily assembled. The permanent magnet is a reliable component because it can retain its magnetism essentially forever if it is isolated from other magnets, power lines, or high temperatures. Modern materials of permanent magnets are resistant to shock or mechanical vibrations if they are not mechanically damaged [49].

**Figure 7:**
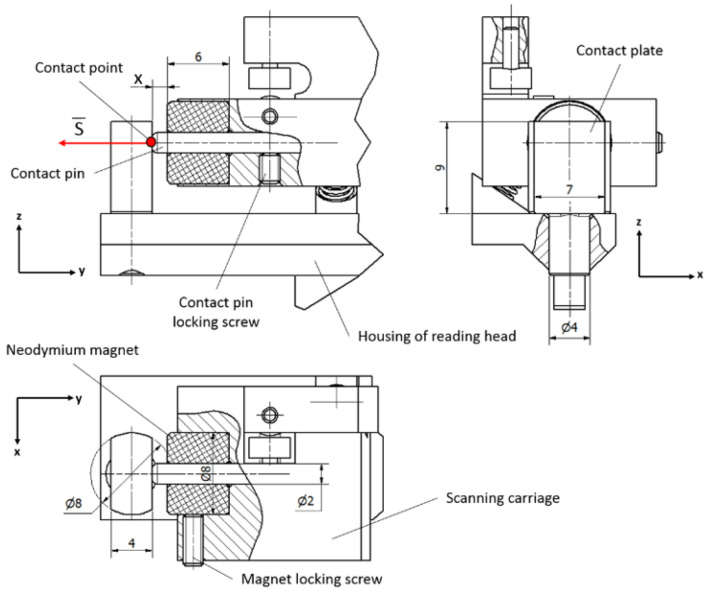
Mechanical construction of contact pair A.

Theoretically, the best performance of the contact pair is reached when both components touch each other at one point only. Taking this fact into account, the permanent magnet pulls the steel plate through an air gap *x* to avoid contact between surfaces of these elements. A mechanical design of contact pair A is shown in Figure 7.

In this construction, the contact steel plate is mounted onto the housing of the reading head and the contact pin, together with the magnet, is placed onto the scanning carriage. Any transition in this pair makes the contact point move over the surface of the plate, which should have precise geometry and be aligned perpendicular to the acting magnetic force *S*.

The size of the air gap should be properly evaluated because the strength of a magnetic field drops off approximately exponentially over the distance. For a circular magnet, the strength of the magnetic field could be calculated from the following equation:

(12) $$F_{field}=\frac{B_{r}}{2}\left(\left(\frac{l+x}{\sqrt{r^{2}+{\left(l+x\right)}^{2}}}\right)-\frac{x}{\sqrt{r^{2}+x^{2}}}\right)$$

where *l* is the length, *r* is the radius of the circular magnet, and *B_r_* is the residual induction. The air gap is indicated by *x*.

**Figure 8:**
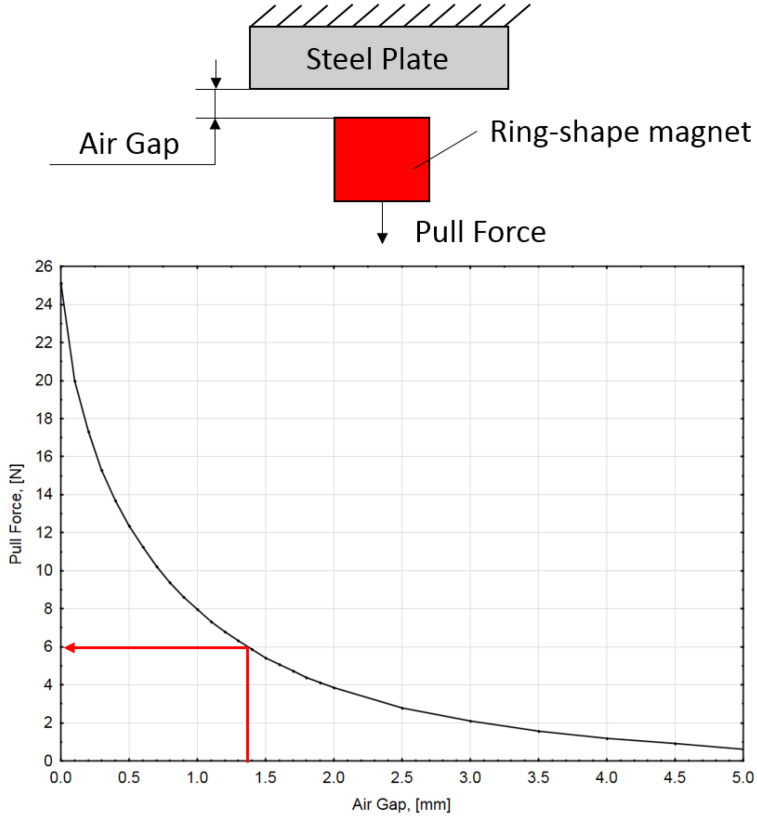
Magnet pulling force vs. distance. (Specified for the ring-shaped permanent magnet used in contact pair A).

The pulling force generated by the magnet is difficult to calculate, so the force dependency from the distance is usually determined experimentally by the manufacturer. Figure 8 shows the pulling force of a ring-shaped neodymium magnet of a selected size in relation to the air gap graph.

According to Equation (11), the acting magnetic force cannot be smaller than ~6 N. If this condition is maintained, the air gap cannot be larger than 1.4 mm. This could be easily achieved by changing the length of the contact pin and fixing it with a locking screw.

#### 3.2.2. Contact Pair B

Unlike the others, contact pair B is placed in the center of the reading head to absorb much more easily oscillations of the suspension. Shorter distances between ball bearings and a contact point will generate less acting torque. In this construction, two hardened 5 mm in diameter pins are kept in contact by two extension springs. One of them is vertically mounted onto the housing of the reading head, while the other one is horizontally placed in the cursor. Any translational or rotational motion of these contact pair elements will always assure connection that is concentrated in a single point.

**Figure 9:**
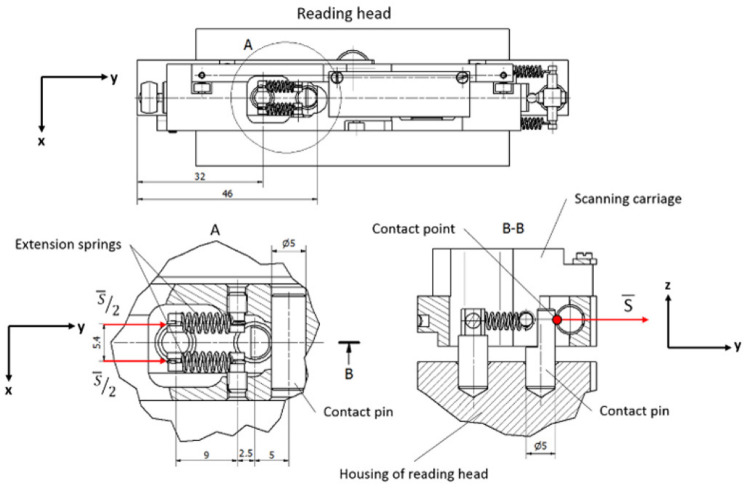
Mechanical construction of contact pair B.

Therefore, two standard-geometry stainless-steel springs are used. The required force per spring is *S/2*. The spring rate of this chosen flexible element is 0.4 N/mm. A mechanical design of contact pair B is shown in Figure 9.

#### 3.2.3. Contact Pair C

**Figure 10:**
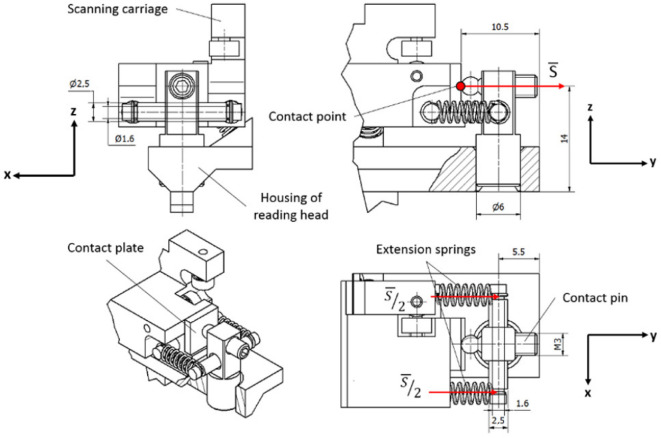
Mechanical construction of contact pair C.

Contact pair C is placed at the end other than pair A. Contact is created between the grinded steel plate and the M3 screw with a hardened bearing ball attached to one end. Differently than in the first pair, the contact plate is fixed on the cursor and the pin is mounted onto the housing of the reading head. The same geometry and rate extension springs as in contact pair B are used. Their tension can be adjusted by screwing the M3 bolt. A mechanical design of contact pair C is shown in Figure 10.

## 4. Experimental Results

The accuracy of an encoder is described as the error between its readout position and its actual position and is determined during the calibration process when the measured values of the tested linear encoder are compared with the reference values, usually given by a laser interferometer. Such a comparison is made in well-defined conditions. The reading head and the aluminum housing of the encoder are accurately mounted onto the technological equipment (comparator). All measurements are performed in a thermal-stable laboratory where the temperature is 20 ± 0.2 °C.

**Figure 11:**
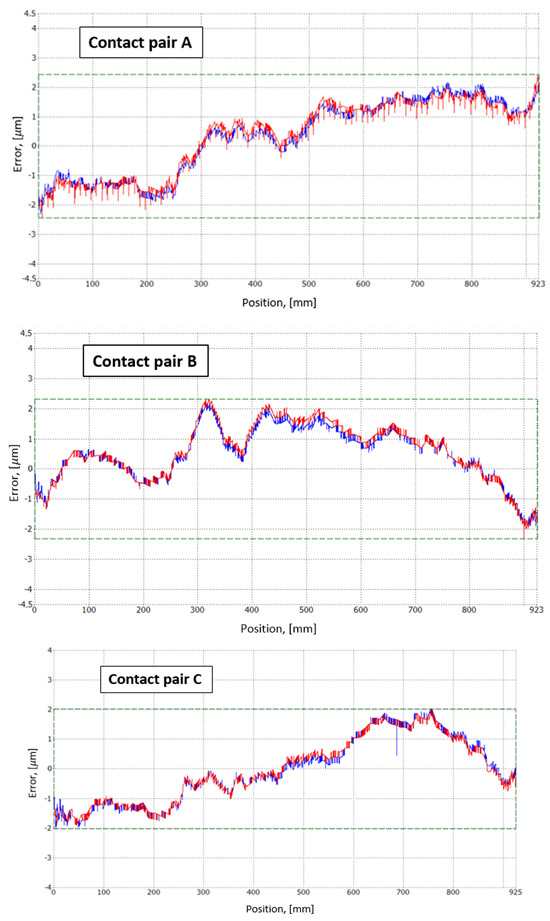
Error of the linear encoder with different mechanical constructions of the experimental reading head. (The graphs show the results of different contact pairs (**A**–**C**). Red and blue lines describe the position measurement errors when the reading head moves forward and backward, respectively).

The designed experimental reading head is manufactured, and its accuracy is tested by JSC “Precizika Metrology” [50]. The same aluminum housing with a 1 m long glass scale is used in all tests to maintain the same conditions and decrease the uncertainty of the comparison process. The results are plotted in three different graphs (Figure 11) for each of the contact pairs and are presented as an error of the linear encoder with different mechanical constructions of the experimental reading head.

The results are plotted in three different graphs (Figure 11) for each of the contact pairs. The identified accuracy values of the reading head with contact pairs A, B, and C are ±2.5 µm/m, ±2.3 µm/m, and ±2 µm/m, respectively. The best result under the well-defined conditions is achieved by the mechanical construction of the reading head with contact pair C.

Assessing Figure 11, the information provided shows that (for contact pair A) there is a significant difference in the error due to forward and backward movement up to 0.9 µm in the range of 200 to 600 mm and the jump values of the regular error occur up to 0.8 µm every 9 mm. The evaluation of contact pair B shows that there is also a significant difference in the error due to forward and backward movement in the range from 250 to 650 mm and that the jump error values occur up to 0.3 µm. Evaluation of contact pair C shows that in comparison with the variants, the error of the forward and backward movement of contact pairs A and B is small and reaches up to 0.4 µm in the range of 450 to 650 mm. Based on the results obtained, it can be seen that the C variant of the contact pair gives the highest stability of the scan head during the measurements.

## 5. Conclusions

Measuring technologies are highly advanced now and continue to improve. Linear displacement measurement devices, such as optical encoders, are constantly upgraded by using new technologies or new optical scanning principles. Generally, the Moiré-effect-based encoders remain the most commonly used in applications such as machine tools and other mechatronic systems.

This work focuses on a specially designed experimental reading head for the Moiré-scanning-principle-based linear encoder. Its mechanical construction is easily changeable, and it could be assembled in three different configurations, i.e., A, B, and C. Each of them consists of particular mechanical elements and is designed to correctly measure linear displacement.

Accuracy tests of all three designs show that mechanical construction C performs the most accurate measurement, with an error of ±2 µm/m. However, designs A and B have similar accuracy, and calibration is done in well-defined mounting and environment conditions. The real encoder’s behavior can be described by more comprehensive analysis, oriented in accuracy and repeatability under different mounting and dynamic conditions.

## Data Availability

The data presented in this study are available on request from the corresponding author.
